# Glycopeptides and -Mimetics to Detect, Monitor and Inhibit Bacterial and Viral Infections: Recent Advances and Perspectives

**DOI:** 10.3390/molecules24061004

**Published:** 2019-03-13

**Authors:** Sandra Behren, Ulrika Westerlind

**Affiliations:** Department of Chemistry, Umeå University, 90187 Umeå, Sweden; sandra.behren@umu.se

**Keywords:** glycopeptides, glycopeptide mimetics, host-pathogen interactions, serodiagnostics

## Abstract

The initial contact of pathogens with host cells is usually mediated by their adhesion to glycan structures present on the cell surface in order to enable infection. Furthermore, glycans play important roles in the modulation of the host immune responses to infection. Understanding the carbohydrate-pathogen interactions are of importance for the development of novel and efficient strategies to either prevent, or interfere with pathogenic infection. Synthetic glycopeptides and mimetics thereof are capable of imitating the multivalent display of carbohydrates at the cell surface, which have become an important objective of research over the last decade. Glycopeptide based constructs may function as vaccines or anti-adhesive agents that interfere with the ability of pathogens to adhere to the host cell glycans and thus possess the potential to improve or replace treatments that suffer from resistance. Additionally, synthetic glycopeptides are used as tools for epitope mapping of antibodies directed against structures present on various pathogens and have become important to improve serodiagnostic methods and to develop novel epitope-based vaccines. This review will provide an overview of the most recent advances in the synthesis and application of glycopeptides and glycopeptide mimetics exhibiting a peptide-like backbone in glycobiology.

## 1. Introduction

Many human proteins are co- or post-translationally modified by mono- or oligosaccharides. Among a number of post-translational modifications, glycosylation is the most abundant form and it has been predicted that nearly 50% of all human proteins are glycosylated. Due to their complexity and structural diversity, carbohydrates and glycoconjugates play crucial roles in many key biological processes. These include pathogenic processes involving viral and bacterial infections. Many viral and bacterial pathogens employ the recognition of carbohydrates displayed on the epithelial surface of cells via pathogenic lectins to penetrate and infect their target organisms. Therefore, the study of these carbohydrate-lectin interactions is important to gain insights into the disease process and progress as well as to develop novel treatment strategies.

The exact functions of the glycans in many of these pathogenic processes are not well understood and their investigation is a demanding task due to technical challenges in glycan analysis, the structural complexity, diversity and inhomogeneity of native glycans and their non-template driven biosynthesis [1]. Another factor is the usually weak intrinsic affinity of single protein-carbohydrate interactions, which in nature is compensated by multiple simultaneous interactions, also known as the multivalency effect [2,3,4,5]. Major efforts to develop strategies to understand and explore the complexity of carbohydrate recognition by pathogenic lectins have been made in recent years. Advances in synthetic carbohydrate chemistry gave access to glycopeptides and glycopeptide mimetics with defined glycan structure and glycosylation sites. Thereby, glycan and glycoconjugate structures can be assembled according to the native compounds, or functional parts of a native compound can be conjugated to mimic the structural function of the respective carbohydrate ligand [6]. The carbohydrate residue can be coupled to either the native peptide, a peptide containing an unnatural amino acid, or a polymer with a peptide-like backbone via for example *C*- or *S*-glycosidic linkages, triazole linkages, oxime or hydrazone ligation, or other unnatural linkages in order to improve the proteolytic stability and specificity of the obtained neo-glycoconjugates while retaining their biological activity. The resulting neo-glycoconjugates are also called ‘glycopeptide mimetics’.

Adhesion of viruses and bacteria to carbohydrate epitopes on the host cell is the initial step in pathogenesis. Therefore, anti-adhesive agents that interfere with the ability of pathogens to adhere to the host cells may represents an attractive approach to fight infectious diseases [7]. Although inhibition of these host-pathogen interactions would be therapeutically significant, the development of effective inhibitors is demanding due to the structural diversity of glycans which includes the configuration, anomeric linkage, ring modification, hydroxyl group masking, charge, hydrophilicity, branching, and conformational properties. An additional challenge in the development of anti-adhesive agents is that most pathogens express more than one type of adhesins that need to be addressed. Thus, synthetic glycopeptides and their mimetics are useful scaffolds for drug development and have become important tools to probe carbohydrate-pathogen interactions.

Another way to fight pathogens is based on glycopeptides that can act as vaccines. During the humoral immune response, T-cells recognize foreign antigens which are processed and presented by major histocompatibility complex (MHC) molecules expressed by antigen-presenting cells (APCs) [8]. For many years, carbohydrates were considered as T-cell independent antigens because of their inability to stimulate T-cell response in their pure form. However, recent research in this field has shown that glycans on peptides can be recognized by T-cells and that recognition of these glycopeptides by T-cells depends on the antigenic glycan as well as the peptide structure [9,10].

Immunization with glycopeptides relies on the elicitation of T-cell help for B-cells recognizing glycans, with promotion of isotype switching from IgM to IgG, and induces the development of memory B-cells as well as T-cell memory [11,12]. The development of point-of-care (POC) serological diagnostics depends on the availability of ligands that specifically are recognized by disease-specific antibodies. Pathogenic glycoproteins should exhibit a large number of different B-cell epitopes available for immune cell recognition, which also results in induction of glycopeptide specific antibodies. Synthetic glycopeptides have become important tools to characterize antibody binding signatures towards glycan and glycopeptide structures, which are present on various pathogens, and are useful to improve serodiagnostic methods and to develop novel epitope-based vaccines.

## 2. Bacterial Infections

The global emergence of multi-drug resistant bacteria is a major threat to human health and the development of new classes of antibiotics that complement the currently available drugs has become an important objective [13]. In the past, novel antibiotics were primarily discovered by screening of natural, semi-synthetic or fully synthetic compound libraries. The screening of natural products has been quite successful as exemplified by the discovery of the penicillins, the cephalosporins, the aminoglycosides, the tetracyclines, the macrolides, and the macrocyclic peptides such as vancomycin and teicoplanin [14]. These groups of highly important antibiotics belong to a large topic which has been described in detail in other recent reports and will therefore not be further discussed in this review [15,16]. During the last years, new generations of these drug classes have emerged by chemical modification and optimization to inhibit a vast number of virulence factors, but it seems unlikely that this can go indefinitely. Therefore, enormous efforts have been made in recent years to design new and efficient scaffolds including proteins [17,18], fullerenes [19], calixarenes [20], nanomaterials [21] and polymers [22] displaying glycans in a multivalent fashion that target anti-adhesive binding events. This review highlights recent advances in the development of glycopeptide and -mimetic scaffolds to study host-pathogen interactions.

### 2.1. Escherichia coli (E. coli)

Uropathogenic *Escherichia coli* (UPEC) is a Gram-negative pathogen that causes urinary tract infections, one of the most common infections, with millions of cases every year [23]. The adhesion of UPEC is mediated by FimH which is a bacterial lectin of type 1 fimbriae (fim) that recognizes α-d-mannosides [24]. Therefore, FimH is an attractive target for the development of anti-adhesive agents [25,26]. X-ray studies have shown that the bacterial FimH lectin possesses a monovalent carbohydrate recognition domain (CRD) that specifically recognizes α-d-mannose [27,28,29]. The carbohydrate binding site is surrounded by two tyrosine residues, Tyr48 and Tyr137, often called the “tyrosine gate” [30]. Monovalent α-mannose residues with a hydrophobic aglycon show increased binding affinity for FimH due to π–π stacking interactions with the aromatic tyrosine residues [31]. This finding can have crucial relevance in the development of potent FimH inhibitors. Even though FimH possesses a monovalent binding site which can bind to only one α-d-mannosyl moiety, binding studies with multivalent carbohydrate ligands have proposed an additional binding site on the lectin [32].

**Figure 1:**
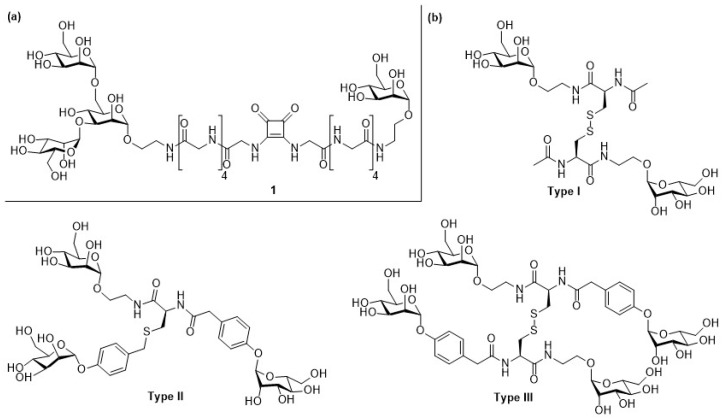
(**a**) The bivalent glycopeptide was assembled by coupling the azido-functionalized mannotrioside and mannoside to pentaglycine spacers, respectively. Both building blocks were subsequently combined via a squaric acid diester linkage; (**b**) Cysteine was used as scaffold to generate glycoclusters with different valencies. Divalent glycoamino acids of type I, trivalent glycoclusters of type II and tetravalent disulfide dimers of type III were synthesized.

In order to verify this hypothesis, the Lindhorst group designed a bivalent mannosylated glycopeptide ligand for evaluation in an anti-adhesion test with type-1 FimH [33]. In search of potential additional carbohydrate-binding sites on FimH, the surface of the bacterial lectin domain was probed by computational docking studies and a monomeric and a trisaccharide mannose ligand were selected as carbohydrate ligands and coupled to a pentaglycine spacer. Finally, the monosaccharide and the trisaccharide building blocks were connected via a squaric acid diester linkage and the binding of obtained glycoconjugates towards FimH was evaluated using anti-adhesion assays (Figure 1a). The results of the binding assays were inconclusive and indicated that determination of the exact number of carbohydrate binding sites on FimH requires further investigation.

Following a different approach, the Lindhorst group used the amino acid cysteine as a scaffold to generate cysteine-based glycoclusters that can act as FimH inhibitors [34]. Here, functionalized mannose moieties were coupled to cysteine to prepare divalent glycoconjugates (Figure 1b). Trimeric or dimerized glycoclusters were generated by modification of the cysteine mercapto group, or by disulfide bond formation. Subsequently, the inhibitory activity of the glycoclusters towards FimH was tested in an inhibition adhesion assay. It was shown that all glycoclusters bound to *E. coli* in the micromolar range and that the ligand multivalency and the contribution of the π–π interactions between the aromatic aglycons with the tyrosine gate had a beneficial impact on the inhibitory potency of the glycoconjugates. The weakest binding potency was observed for the divalent type I cluster mannoside (IC_50,type I_ = 240 µM), lacking any aromatic aglycon moiety, whereas the trivalent type II cluster mannoside performed best in the assay (IC_50,type II_ = 30 µM).

**Figure 2:**
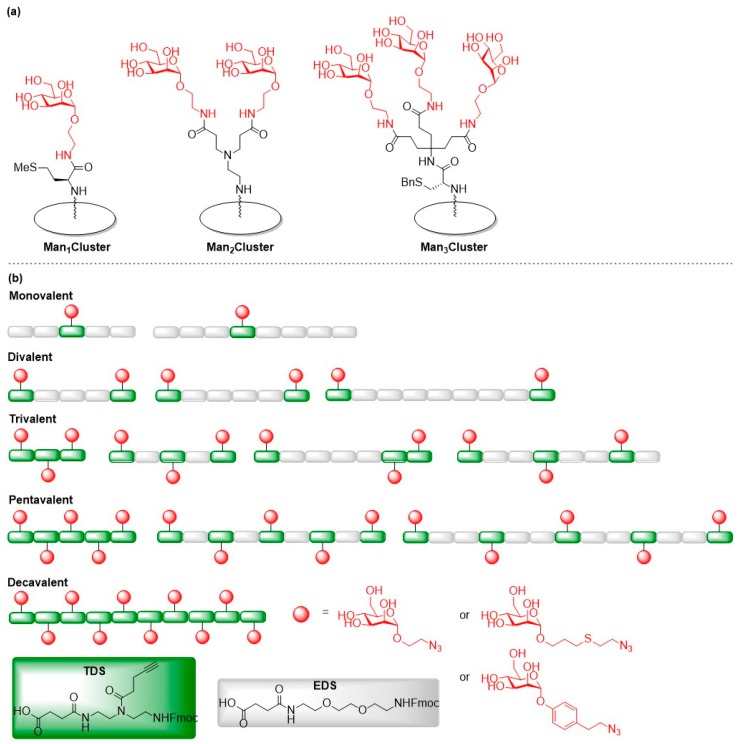
(**a**) Glycoarrays presenting mono-, di- and trivalent mannoside ligands; (**b**) Overview of linear glycomacromolecules carrying α-d-mannose ligands with varied ligand valency and interligand spacing.

To further explore the carbohydrate-lectin interaction during the fimbriae-mediated bacterial adhesion process, glycoconjugate microarrays were used to investigate the impact of different concentrations and valences of mannose ligands on the stickiness of a glycosylated surface [35]. A of mono-, di- and trivalent mannoside conjugates, Man_1_Cluster, Man_2_Cluster and Man_3_Cluster, were synthesized by coupling azidoethyl mannosides to the respective mono-, di- and trifunctional carboxylic acid derivatives via Staudinger ligation (Figure 2a). The glycoconjugates were immobilized on microtiter plates in different concentrations and the glycoarrays were incubated with fluorescently labeled type 1 fimbriated *E. coli*.

The inhibitory activity of the monovalent derivative was reduced with decreasing concentration of the glycoconjugates (from 15 mM to 2 mM) which is accompanied by a reduced ligand presentation for FimH-mediated adhesion. Surprisingly, the opposite effect was observed for binding of the multivalent glycoclusters to FimH, which is related to a high local concentration of carbohydrate ligands at lower concentrations of glycoconjugates present on the array. This effect proves to be beneficial for bacterial adhesion. The authors concluded, that the differences in stickiness of the glycoarray surfaces depends on the ligand density which is based on the glycocluster structures.

Based on the results of a previous study [36], the Hartmann group generated a library of mannosylated glycomacromolecules to test in binding assays with *E. coli* [37]. Mannosylated glycooligomers with three varying structural parameters—the valency and the spatial arrangement of the mannose ligands as well as the chemical nature of the linker that conjugated the ligand to the peptide backbone—were prepared via alternating coupling of two tailor-made building blocks EDS and TDS using solid phase polymer synthesis (SPPoS) (Figure 2b). EDS was used to introduce a diethylene glycol unit as spacer in the backbone of the oligomer, whereas TDS exhibits an alkyne side chain for subsequent coupling of azido-functionalized mannose ligands via on-resin copper mediated azide alkyne cycloaddition reactions (CuAAC). The obtained glycooligomers were tested in binding studies with ConA which was used as model lectin to pre-select potent binders for subsequent binding studies with FimH using isothermal titration calorimetry (ITC) experiments. Glycomacromolecules containing an aromatic aglycon showed enhanced affinity to ConA. These findings parallel earlier studies with the bacterial lectin FimH where increased affinity towards FimH was explained with the favorable π–π-interactions with the tyrosine gate [36]. Therefore, these glycooligomers were chosen for a bacterial adhesion-inhibition test to determine their inhibition efficiency on type 1 fimbriae-mediated bacterial adhesion. In contrast to the former experiment, where the multivalent presentation and the variation of the spatial arrangement of the mannose ligand had a strong impact on the binding affinity to FimH, no correlation between the valency and spatial arrangement, and the inhibition efficacy of the glycooligomers could be found in this study. The authors assumed that the inhibitory activity of the glycomacromolecules is related to the sterical shielding of the non-binding components of the glycopeptides due to their adoption of a coiled conformation in solution.

### 2.2. Pseudomonas aeruginosa

*Pseudomonas aeruginosa* (PA) is a Gram-negative bacterium that causes serious and chronic infection of the respiratory and/or urinary tracts, skin and eyes especially of immune-depressed patients leading to high morbidity and mortality [38,39,40]. Furthermore, PA possesses the ability to develop antibiotic resistance by forming biofilms. The PA lectins LecA and LecB are tetrameric adhesins that are involved in the bacterial biofilm formation and specifically recognize and bind to d-galactosides and l-fucosides, respectively [41,42]. Thereby, LecA shows a preference for α-linked terminal galactose units on longer glycans [43]. On the other hand it was observed, that single galactose moieties such as phenylated β-d-galactosides were more potent inhibitors than the corresponding α-d-galactoside derivatives [41]. LecB exhibits a preference for α-l-fucosides over β-l-fucosides [44]. Therefore, glycoconjugates interfering with LecA or LecB binding have the potential to lead to the development of new anti-biofilm and anti-adhesion therapies.

**Figure 3:**
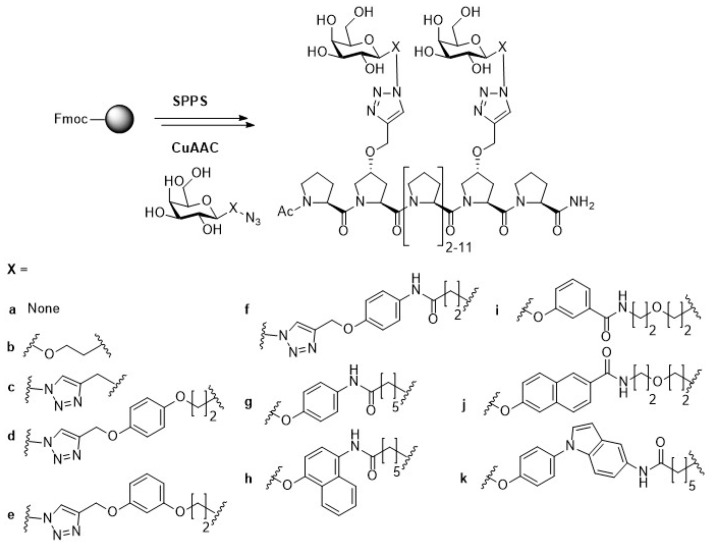
Design of polyproline glycopeptides with varying interligand spacing and various galactose ligands.

The distance between the two closest binding sites of the tetrameric LecA is about 26 Å [45]. An effective LecA inhibitor that fits the binding sites on LecA was designed using the well-defined polyproline helix II structure to create bivalent scaffolds presenting β-d-galactosides in a controlled spatial arrangement (Figure 3) [46]. In these peptide scaffolds, the inter-ligand distance can be altered as required. Polyproline peptides containing alkyne-functionalized prolines with spacings of 9, 18, 27, and 36 Å for subsequent conjugation with azido-modified galactose moieties were prepared by solid phase peptide synthesis (SPPS).

The binding to LecA was evaluated by a surface plasmon resonance (SPR) assay. The experimental data showed that the optimal spacing between the galactose ligands to fit LecA is three helical turns (27 Å). The chosen scaffold was then modified with different galactose derivatives to further optimize the binding efficacy towards LecA. Galactose-derived ligands bearing triazole and/or aromatic rings as well as other aromatic variants such as *para*-phenyl and naphthyl galactosides were generated. SPR assays showed that the glycopeptides carrying spacers a, b, c, g, i, j and k bound to LecA in the nanomolar range (K_d_ = 136–442 nmol for SPR assay performed with 5% DMSO), whereas glycopeptides bearing the other spacers showed no binding activity. Most of the ligands bearing an aromatic aglycon exhibited better binding activity (K_d,arom_ = 136–278 nmol) than the triazole galactoside (K_d,c_ = 363 nmol). The glycopeptide containing the aromatic aglycon g was the best binder (K_d,g_ = 136 nmol). It was reported that ligands presenting an β-aromatic aglycon significantly increase the affinity toward LecA by initiating CH–π interaction with the histidine residue at the binding site of LecA [47,48]. In short, this new multivalent polyproline peptide is a promising scaffold for LecA inhibitor development.

**Figure 4:**
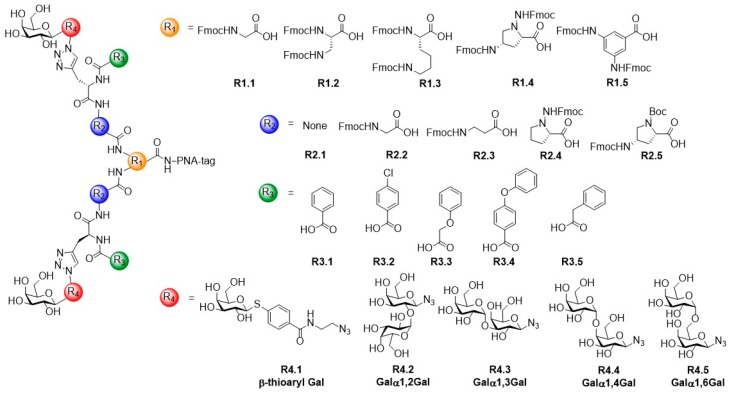
Design of a library consisting of bivalent galactosylated glycoconjugates with varying structural parameters to bridge the neighboring binding sites of LecA.

In another study, a library of glycoconjugates containing galactose ligands with a spacing of ca. 30 Å, and an aryl substituent in the linker was prepared to fit the dimeric geometry and binding preferences of LecA [49]. Galactosylated glycoconjugates with four varying structural parameters were synthesized using peptide nucleic acid (PNA)-encoded synthesis via *SPPS*: (i) different branching geometries and lengths (R1.1–1.5), (ii) diverse spacer groups with different lengths and geometries (R2.1–2.5), (iii) different aryl groups in the linker moiety (R3.1–3.5) and (iv) different permutations of 1,2- 1,3- 1,4- and 1,6-α-galactose moieties (R4.2–4.5) as well as the β-thioarylgalactose (R4.1) ligand (Figure 4). After cleavage of the glycoconjugates from the solid support, each compound was linked to an unique PNA sequence that encodes its synthetic structure and was subsequently hybridized on a microarray carrying complementary sequences [50]. This way, screening of the PNA-tagged library with LecA was carried out using the glycoconjugate library as a mixture to select the best binder followed by analysis of the PNA-tag.

A binding preference for the derivative carrying the β-thioaryl galactose ligand and the phenoxyacetate group in the linker unit (R1.3–2.5–3.3–4.1) was observed at a LecA concentration of 5.6 nmol. A binding assay additionally including the respective monovalent derivatives showed binding preferences of the bacterial lectin towards glycoconjugates with divalent ligand presentation over the monovalent variants at lower ligand concentration and surface density. Only at high ligand concentrations, the ligand density was sufficiently high for LecA to interact with multiple monovalent ligands on the array. The chosen divalent ligand was resynthesized substituting the PNA tag for an arginine residue and its affinity to LecA was determined by ITC. A strong binding affinity to the lectin in the nanomolar range was observed. Then, the bivalent compound was co-crystallized with LecA. The obtained structure confirmed previous findings of the thioaryl ring establishing a CH–π interaction with the histidine residue of LecA [47,48]. Additionally, the triazole and the proline rings were stabilized by a water-bridged interaction either involving the side chains of His50, Tyr36 and Asp47 residues at the LecA binding site, or a sulfate ion. In a final experiment, the potency of the chosen bivalent ligand to block bacterial entry into human lung epithelial cells was determined using an Amikacin protection assay. Cultures of *P. aeruginosa* were first incubated with the obtained glycoconjugates and subsequently with human lung epithelial cells. Extracellular bacteria were inactivated by treatment with Amikacin, which is an aminoglycoside antibiotic with bactericidal activity. The cells were lysed and the bacterial invasion of Amikacin-survived *P. aeruginosa* was quantified comparing the relative ratio of intracellular bacteria found in glycoconjugate pre-treated cells to untreated lung epithelial cells. The bivalent glycoconjugate bearing the PNA-tag was able to reduce the internalization of *P. aeruginosa* by 83% at 50 nmol. Additionally, the respective derivate without the nucleic acid tag as well as oligomers of this ligand derived from its hybridization to different DNA or PNA scaffolds were evaluated and a decreased uptake of *P. aeruginosa* by 30–86% was observed.

**Figure 5:**
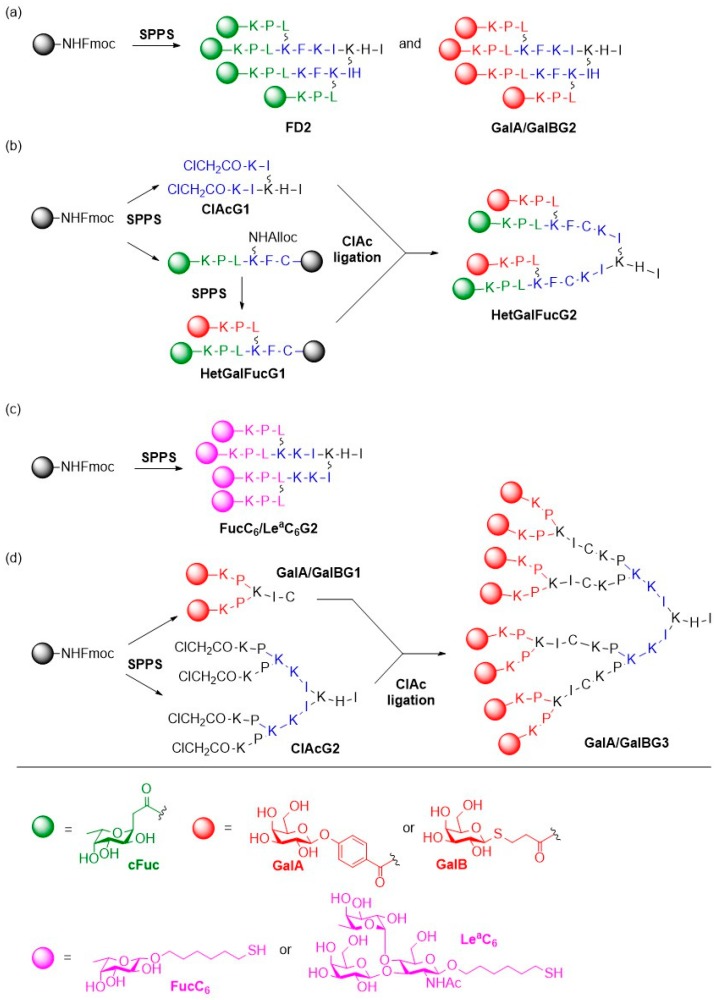
(**a**) Structure of the fucosylated FD2 and galactosylated GalA/GalBG2 glycodendrimers; (**b**) Synthesis of the heteromultivalent fucose- and mannose-functionalized glycodendrimer via SPPS and chloroacetyl cysteine thioether ligation; (**c**) Preparation of homomultivalent FucC_6_G2 and Le^a^C_6_G2 dendrimers; (**d**) Synthesis of octavalent galactosylated glycodendrimers.

The group of Reymond et al. reported tetravalent glycopeptide dendrimers (FD2) functionalized with α-l-C-fucosides [51], or phenylgalactoside (GalA) or thiopropylgalactoside (GalB) ligands [48] at the end of a second generation (G2) peptide dendrimer scaffold as inhibitors of LecB and LecA, respectively (Figure 5a). These studies showed that the glycodendrimers exhibited good inhibition activity towards the respective lectin and induced partial dispersion of *P. aeruginosa* biofilms. In agreement with the before mentioned findings, glycodendrimers containing an aromatic aglycon (GalA) showed stronger binding than their GalB analogs that carry an aliphatic chain as aglycon due to the beneficial CH–π interaction between the aromatic group and the His50 residue of LecA. During the last years, this group has developed different strategies to further optimize these structures, for example by incorporating d-amino acids into the glycodendrimer FD2 to provide resistance against proteolysis [52].

Following an original approach, the Reymond group used SPPS and chloroacetyl cysteine thioether (ClAc) ligation to prepare heteroglycodendrimers to target both LecA and LecB (Figure 5b) [53]. Heteromultivalent glycodendrimers carrying fucosyl and galactosyl groups were synthesized to determine if a scaffold that target both lectins might show enhanced binding potency. The binding affinities of different gylcodendrimers to *P. aeruginosa* lectins LecB and LecA were evaluated by ITC. The heteromultivalent compounds showed decreased binding activities to both lectins (K_d,Het_ = 121–327 nmol for LecB and K_d,Het_ = 75–340 nmol for LecA) compared to their corresponding homomultivalent derivatives FD2, GalAG2 and GalBG2 that bind to LecA and LecB in the lower nanomolar range (K_d,FD2_ = 66 nmol, K_d,GalAG2_ = 2.5 nmol, K_d,GalBG2_ = 40 nmol). Biofilm inhibition activities of the heteromultivalent glycodendrimers were investigated and good biofilm inhibition could be observed for all glycodendrimers except for some of the GalB containing heteroglycoclusters. Most of these tested compounds were also able to disperse already formed biofilms with the exception of a few heteroglycoclusters that were toxic at their biofilm inhibition concentration. Then, homomultivalent Lewis^a^ and thiohexyl (C_6_)-fucose dendrimers were prepared based on the potent FD2 dendrimer (Figure 5c) and showed good binding affinities to LecB in the nanomolar range (K_d,FucC6G2_ = 121 nmol, K_d,Le_^a^_C6G2_ = 39 nmol) as measured by ITC. Biofilm inhibition and dispersal potencies in the same range as for FD2 were observed for both glycodendrimers, with a slightly enhanced biofilm inhibition in case of the thiohexyl-fucose dendrimer.

In another report, generation G3 and G4 derivatives of GalA/BG2 glycodendrimers with higher valencies were prepared by the above mentioned chloroacetyl cysteine (ClAc) thioether ligation to increase the binding affinity towards LecA as well as the biological activity (Figure 5d) [54]. Binding studies with LecA by hemagglutination inhibition assays and ITC showed that the positive impact on the relative binding affinity per galactose ligand which was observed for the tetravalent G2 dendrimers only partially applied to the octavalent G3 dendrimers. Surprisingly, a decrease of the relative binding affinities was observed for G4 dendrimers. Similar results were obtained for *P. aeruginosa* biofilm inhibition and dispersal assays, with G3 dendrimers showing comparable potencies in biofilm inhibition to G2 dendrimers and only slightly better activities in biofilm dispersal. Consequently, the search for improved FD2 and GalA/BG2 derivatives must be continued.

**Figure 6:**
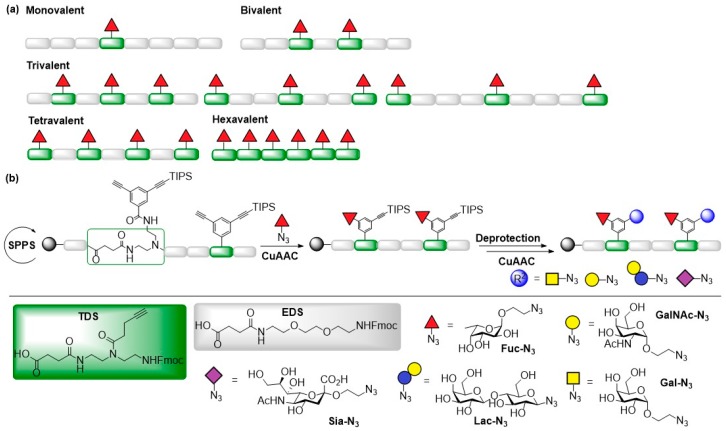
(**a**) Structure and preparation of building blocks TDS and EDS and fucosylated glycooligomers; (**b**) Synthesis of heteromultivalent glycooligomers with introduction of different carbohydrate ligands by consecutive CuAAC on solid support.

In one study, fucosylated glycooligomers were synthesized and their inhibition potential towards LecB of *Pseudomonas aeruginosa* and biofilm formation was explored. The sequence-controlled α-l-fucose-functionalized glycooligomers were assembled via alternating coupling of above mentioned building blocks EDS and TDS using SPPoS (Figure 6a) [55]. Fucosylated glycooligomers with valencies ranging from one to six carbohydrate ligands, and trivalent derivatives with one, two, or three EDS spacers between the fucose-functionalized TDS building blocks were prepared. SPR experiments and enzyme-linked lectin assays (ELLA) were performed to study the inhibitory activity of the generated fucosylated glycooligomers on LecB. The results of both experiments showed that all glycooligomers bound to LecB in the nanomolar range (IC_50_ = 22–130 nmol) with the hexavalent glycooligomers being the best binder, and that higher valencies lead to increased inhibitory effects indicating higher avidity. While binding of the glycooligomers towards LecB was enhanced with increasing number of ligands in the SPR experiment, the inhibition of LecB was only enhanced for glycooligomers with three or more fucose ligands in ELLA. An increasing number of EDS spacers between the TDS building blocks only lead to a slightly decreasing inhibitory effect in SPR experiments indicating a minor impact of ligand spacing on the binding potency. The ELLA experiments showed highest and lowest biding activity towards LecB for the derivative with two and one EDS building blocks, respectively. Finally, the fucosylated glycooligomers were probed for their potency to inhibit biofilm formation of *P. aeruginosa* in vivo. The test confirmed that all tested candidates exhibited inhibitory activity and showed reduction of the biofilm formation in a range of 15–20%.

In a similar study, heteromultivalent glycooligomers bearing fragments of histo-blood group antigens (HBGA) and human milk oligosaccharides (HMO) were prepared and their inhibitory activity towards the bacterial lectin LecB was determined (Figure 6b) [56]. Precision glycomacromolecules carrying a free and a TIPS-protected alkyne group were assembled by SPPoS. Subsequently, different carbohydrate ligands were attached by using an orthogonal coupling strategy and sequential coupling of the carbohydrate moieties via CuAAC. The inhibitory potencies of the obtained heteromultivalent glycooligomers towards LecB from *Pseudomonas aeruginosa* were determined.

Enhanced inhibition of LecB in the nanomolar range (IC_50_ = 35–76 nmol) was reported for all tested glycocooligomers. Similar binding results for different heteromultivalent glycooligomers indicated that the second non-fucose ligand had no major impact on the overall binding efficiency. Interestingly, no significant difference in binding between a homomultivalent fucosylated variant exhibiting a total of four fucose ligands and the heteromultivalent derivatives presenting only two fucose moieties was observed. These findings are not in line with the previously mentioned study where binding to LecB was enhanced with an increasing number of fucose ligands presented on the oligomeric backbone. The authors concluded, that further studies to investigate the participation of different carbohydrate ligands in LecB binding are required.

The group of Renaudet and Dumy have used cyclopeptide scaffolds in previous work to construct synthetic vaccines [57,58,59]. Cyclopeptides are useful scaffolds for multivalent presentation of glycans and possess numerous advantages: (i) their non-immunogenicity, (ii) the possibility to present ligands in a well-defined spatial arrangement, (iii) the possibility to combine different epitopes or glycan structures, (iv) their improved resistance against proteolytic degradation [60].

**Figure 7:**
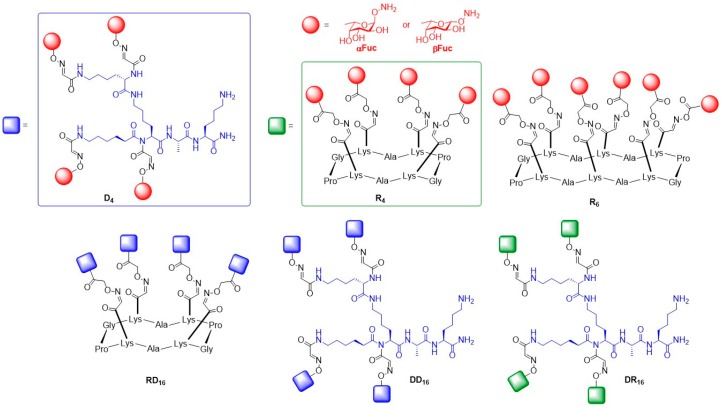
Structures of tetra- (R_4_ and D_4_), hexa- (R_6_), and hexadecavalent (RD_16_, RR_16_, DR_16_, DD_16_) fucosylated glycoclusters.

Based on the concept of template-assembled synthetic proteins, this group designed Regio-selectively Addressable Functionalized Templates (RAFT). These RAFTs are cyclopeptide scaffolds composed of an anti-parallel β-sheet constrained by two adjacent proline-glycines as β-turns. Additionally, they are stabilized into a locked and rigid conformation by intramolecular hydrogen bonds. Lysine side chains are oriented on both sides of the scaffold to provide multivalent binding sites for orthogonal functionalization. The cyclopeptide scaffold was prepared by SPPS of the linear peptide sequence with subsequent cyclization in solution. By applying this strategy, Renaudet and Dumy aimed to develop new multivalent glycoconjugates with higher affinity for LecB [61]. Novel glycoclusters and glycodendrimers displaying α- or β-fucosides to additionally explore the influence of the anomeric linkages towards lectin binding were prepared and their binding potency toward LecB were tested by using ELLA and ITC. Tetra- and hexavalent RAFT cyclopeptides R4 and R6 and a flexible lysine-based dendron D_4_ were synthesized (Figure 7). Additional 16-valent structures were generated by conjugating both tetravalent scaffolds with each other in all possible combinations to give RR_16_, RD_16_, DD_16_ and DR_16_. The competitive ELLA tests showed that tetravalent and hexavalent presentation of both fucose anomers were not resulting in a significant binding enhancement towards LecB. Strong inhibition effects in the nanomolar range were observed for the 16-valent glycoconjugates with favorable binding of α-l-fucosides towards LecB (IC_50_ = 0.6–11 nmol) over β-l-fucosides (IC_50_ = 51–109 nmol) with αFuc-RD_16_ being the best binder (IC_50,αFuc-RD16_ = 0.6 nM). ITC experiments were performed to determine the stoichiometry and thermodynamic contribution of the binding in solution. The results indicate that each glycoconjugate can bind up to six monomers of lectins.

### 2.3. Burkholderia cepacia

Members of the *Burkholderia cepacia* complex (Bcc) are another class of opportunistic Gram-negative related bacteria that cause severe respiratory infections in cystic fibrosis patients [62,63]. A member of this group is *Burkholderia cenocepacia* and due to its antibiotic resistance and ability to form biofilms, the development of efficient drugs has become a major focus in this field of research [64]. The genome of *B. cenocepacia* contains three LecB-like genes [65]. One of the expressed proteins is BC2L-A (*B. cenocepacia* lectin A), which is a calcium-dependent homodimer consisting of 13.8 kDa proteins with a β-sheet core structure. BC2L-A binds to α-d-mannosides with high affinity [66,67].

Using the abovementioned strategy, mannose-functionalized glycoclusters and glyco-dendrimers with valencies of four, six and 24 ligands were synthesized by the group of Renaudet and Dumy in order to evaluate the impact of valency and tridimensional structure on the binding affinity of the glycoclusters towards BC2L-A [68].

**Figure 8:**
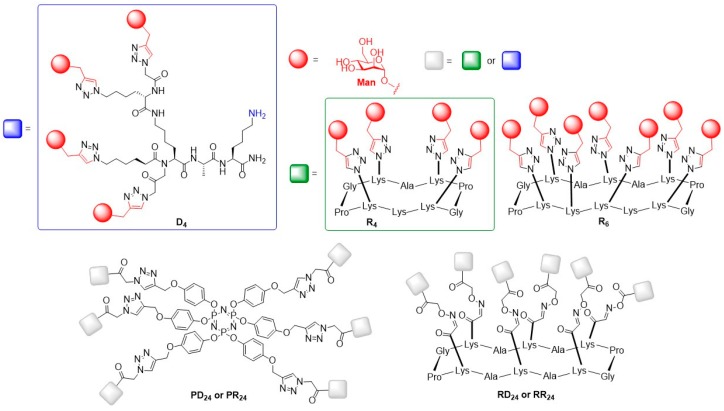
Structures of tetra- (R_4_ and D_4_), hexa- (R_6_), and 24-valent (PR_24_, PD_16_, RR_16_, RD_16_) mannosylated glycoclusters.

In this study, tetra- and hexavalent RAFT cyclopeptides R_4_ and R_6_, a flexible lysine-based dendron D_4_ and a phosphazene core P_6_ were designed for multivalent presentation of mannose ligands (Figure 8) [68]. Based on these structures, different derivatives were prepared to give 24-valent glycodendrimers. The binding potencies of the obtained glycoclusters and glycodendrimers exhibiting varying flexibilities and architectures to lectin BC2l-A were evaluated by ITC.

The binding efficacy of the mannosylated glycodendrimers to BC2l-A increased with an increasing number of ligands present on the scaffolds from a higher nanomolar (K_d,Man4_ = 448–481 nM, K_d,Man6_ = 199–368 nM) to a lower nanomolar range (K_d,Man24_ = 51–256 nM) with construct PD24 being the best binder (K_d,PD24_ = 51 nM).

A significant difference in the binding towards BC2l-A was observed for the tetravalent derivatives R_4_ and D_4_ as well as the hexavalent variants R_6_ and P_6_ which were built on different scaffolds: The more rigid cyclopeptide scaffolds were favored in both cases most probably due to a better fitting geometry for binding to BC2L-A. The evaluation of the 24-valent glycodendrimers showed that the core scaffolds had no major impact on the binding affinity. Glycodendrimers built on the same core structure with lysine-based dendrons as peripheral scaffolds showed enhanced binding compared to scaffolds with ligand presentation through cyclopeptides. This effect was related to the flexibility of the dendrons, which could facilitate the binding of the ligands to the carbohydrate binding site of the lectin.

**Figure 9:**
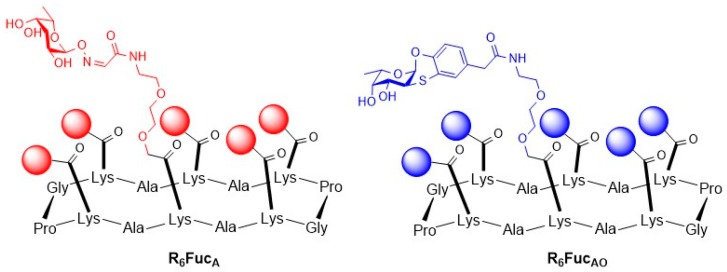
Structure of the hexavalent glycoconjugates carrying aminooxylated fucose or aryl fucose ligands, respectively.

The same group also prepared fucose-functionalized hexavalent glyco-peptides as inhibitors to specifically target fucose binding lectins from *Pseudomonas aeruginosa* and *Burkholderia ambifaria* [69]. *Burkholderia ambifaria* is a further member of the above mentioned *Burkholderia cepacia* complex [70]. The *B. ambifaria* lectin BambL consist of repeat sequences of 40 amino acids forming β-sheets and is formed upon trimerization of two-repeat peptides. BambL binds six fucose residues with high affinity. In this study, fucose-functionalized hexavalent cyclopeptide scaffolds build from 14 amino acids were designed and binding studies with BambL and LecB were performed by ITC (Figure 9). 

The cyclopeptide was synthesized on a solid support with subsequent introduction of a PEG-linker and two different non-natural α-l-fucose moieties, an aryl (Fuc_A_) and an aminooxylated fucoside (Fuc_AO_). Then, the ability of the hexavalent cyclopeptides to inhibit BambL and LecB was evaluated in ITC experiments. Whereas both hexavalent fucose derivatives bind to BambL in a lower nanomolar range (K_d,Fuc(A)_ = 13.8 nM, K_d,Fuc(AO)_ = 16.8 nM), no binding of R_6_Fuc_A_ towards LecB was observed. Compound R_6_Fuc_AO_ binds to LecB in a nanomolar range (K_d,Fuc(AO)_ = 165 nM).

### 2.4. Mycobacterium tuberculosis

In addition to generate efficient strategies to fight bacterial pathogens, another important approach is to develop novel serological diagnostic methods, which enable the search for epitopes that are specifically recognized by disease-specific antibodies. While serological tests have been shown to be effective for the diagnoses of bacterial infections, the serodiagnosis of tuberculosis (TB) remains challenging. *Mycobacterium tuberculosis*, a intracellular pathogen that is a major cause of morbidity and mortality worldwide, is a causative agent of TB [71]. The hydrophobic and complex cell wall of *M. tuberculosis* plays a crucial role in its ability to evade the host immune system [72]. A crucial drawback in the development serodiagnostic methods relies on the fact that infection with *M. tuberculosis* results in the activation of multiple T cell subsets that recognize a great number of different antigens. This results in the induction of indistinguishable antibodies which makes the serodiagnosis of TB difficult. Lipoarabinomannan (LAM) is one of the most abundant glycolipids in the bacterial cell wall and consists of a mannan backbone modified with arabinose moieties linked to a phosphatidylinositol unit [73]. Because of its ability to induce high antibody titers upon infection, it represents an interesting antigen for diagnostic platforms [74].

**Figure 10:**
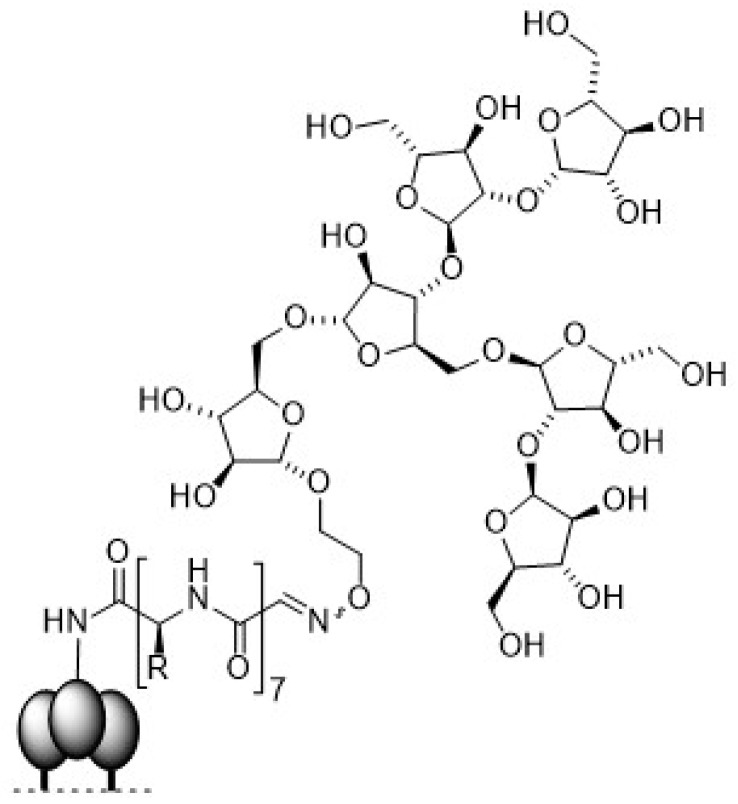
Heptapeptide libraries were displayed on the M13 phage. The glycophage libraries were obtained by periodate oxidation of the *N*-terminal serine residue on the backbone of the randomized heptapeptides and subsequent oxime ligation with Ara_6_ ligands.

A method to identify highly specific and selective ligands for anti-LAM antibodies using phage display of glycopeptides bearing a glycan recognition motif was introduced by the Derda group (Figure 10) [75]. A glycopeptide-presenting phage library containing a hexaoligosaccharide arabinose ligand (Ara_6_) was generated by conjugation of the carbohydrate ligand to the oxidized *N*-terminal serine residue of the heptapeptides via oxime ligation.

After deep sequencing and differential enrichment analysis using the CS-35 antibody, a monoclonal mouse IgG that strongly binds to Ara_6_ [76], 80 potential glycopeptide binders were identified. This glycopeptide library was synthesized on a patterned Teflon-impregnated paper [77] and further evaluated in a microarray binding assay by incubation with tetramethylrhodamine isothiocyanate (TRITC) labeled CS-35 antibodies. The experiment identified Ara_6_-DAHATLR, Ara_6_-ANSSFAP, and Ara_6_-TTYVVNP as the most promising ligands. These findings were also confirmed by determination of the binding constants via ESI-MS experiments. In order to explore the ability of the glycopeptides to discriminate between different anti-LAM antibodies, selected glycopeptides were coupled to multivalent BSA conjugates via squarate ligation. Low (2–4), medium (5–9), or high (>9) loadings of glycopeptides per BSA molecule were obtained and the binding specificities to antibodies CS-35 and 906.4321, which is another anti-LAM monoclonal antibody, were investigated using microarray binding assays. It was observed that the valency of the ligand presentation was critical for specificity. High-valency BSA-glycopeptide conjugates could not discriminate between CS-35 and 906.4321. In contrast, constructs with lower valencies exhibited up to 19-fold selectivity for CS-35 over 906.4321. In conclusion, the glycopeptide ligands were able to distinguish the antibodies with 19-fold increased selectivity and the applied strategy to identify antigens with enhanced affinity and specificity towards a specific anti-LAM antibody, but not towards another closely related antibody is a promising approach to increase the specificity of TB serodiagnostic tests.

## 3. Viral Infections

Antiviral drugs have in later years greatly improved the survival rate especially of human immunodeficiency virus type-1 (HIV-1) infected patients. However, the usually high costs of these drugs, together with the emergence of resistance, make a preventive vaccine an attractive long-term solution to target viral infections. In order to broaden our knowledge of the immunity to viral glycans and glycoproteins, extensive efforts have been made to identify immunogenic glycans and glycopeptide epitopes. Viral envelope glycoproteins contain *N*- and *O*-glycosylation sites with large *N*-glycans consisting of at least eight monosaccharide units, or *O*-glycans that range from one to five monosaccharide building blocks and represent major targets for antibodies that bind and inhibit the viral pathogens [78]. Since the amino acid sequence of the viral glycoproteins is specified by the viral genome, the glycoproteins are immunologically “non-self” and immunogenic.

On the other hand, viral glycans are synthesized and glycosylated by the same glycosylation machinery as are the normal glycoproteins of a human cell and are therefore neither immunogenic nor antigenic [79,80]. Specific immune recognition where both, the glycan and the peptide backbone, contribute to the binding epitope plays a crucial role for the development of immunotherapy and immunodiagnostics. For this reason, neutralizing antibodies that are directed to the glycopeptide epitopes (neutralization epitopes) of the viral glycoprotein could be of particular interest for vaccine development.

### 3.1. Human Immunodeficiency Virus Type-1 (HIV-1)

HIV-1 is the cause of AIDS, a disease which still causes serious global health problems [81]. Despite enormous efforts in the vaccine development, a convincingly effective HIV vaccine is still missing. In order to evade the immune surveillance, the HIV develops numerous defense mechanisms such as frequent sequence variation and heavy glycosylation of the viral envelope glycoproteins [82]. The trimeric HIV-1 envelope protein (Env) is responsible for host cell recognition and subsequent entry of the virus into the cytoplasm [83]. Env is expressed as a gp160 precursor that is proteolytically cleaved into heterodimers of a surface-exposed glycoprotein, gp120, and a transmembrane glycoprotein, gp41. The outer envelope protein gp120 subunit is relevant for the adsorption of virions to chemokine and CD4 receptors on the host cell and has a highly variable surface that includes five variable loops (V1–V5) [84]. The number of conserved *N*-linked glycosylation sites of gp120 ranges from 18–33, with a median value of 25 [85], and include complex or high-mannose type glycans [86,87,88]. The transmembrane envelop glycoprotein gp41 possesses four conserved *N*-glycans and mediates the fusion of the virus and host cell membranes [89].

**Figure 11:**
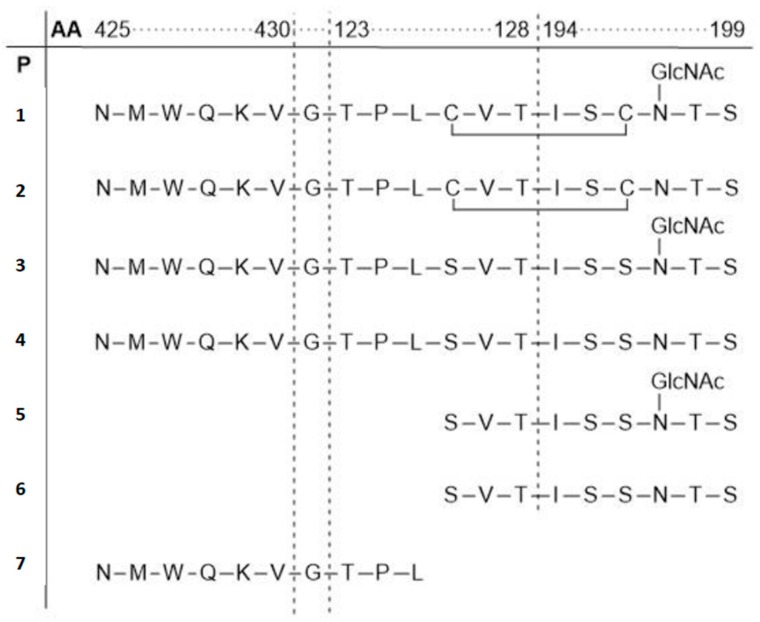
Overview of synthetic gp120 *C*-terminal amidated peptides and glycopeptides prepared by the Meyer group. The amino acid numbers are assigned to their position in the gp120 of HIV-1.

The Meyer group prepared gp120-based peptides and glycopeptides to investigate the impact of glycosylation of gp120 at the N197 position on binding of gp120 to CD4 by SPR and saturation transfer difference NMR spectroscopy (STD) [90]. The peptides were assembled by microwave-assisted SPPS. Based on the amino acid sequence of gp120, linear and cyclic glycosylated and non-glycosylated peptides **P1**–**4** and **P7** were synthesized by combining three noncontiguous peptides according to their position in the crystal structure of the complex gp120–CD4 (Figure 11). Additionally, two linear peptides **P5**–**6** lacking the primary binding motif NMWQKVGTPL were prepared. Binding affinities of the obtained gp120 peptides towards CD4 were determined by SPR.

Peptides **1**–**4** bound to CD4 in the micromolar range. Preferred binding to glycosylated peptides **P1** and **P3** (K_d,glyc_ = 19 and 450 nM, respectively) over the corresponding non-glycosylated derivatives **P2** and **P4** (K_d,non-glyc_ = 83 and 540 nM, respectively) was observed. The binding affinities decreased for peptides lacking the NMWQKVGTPL binding motif. Additionally, the results indicate that the cyclic structure is another important factor that enhances the binding affinity. STD NMR analysis of the binding epitope showed that the GlcNAc moiety interacts primarily at its *N*-acetyl group with CD4. These findings lead to new concepts in CD4 inhibitor development.

Since the *N*-glycans are assembled by the host glycosylation machinery, they are considered as ‘self’ and are therefore only weakly immunogenic. The high density of glycans generates a so called ‘glycan shield’ that usually interferes with antibody recognition. Nevertheless, some of the most potent broadly neutralizing antibodies (bNAbs) from HIV-infected patients have evolved to recognize epitopes on the Env that are formed by these glycans, including 2G12, PG9, PG16, PGT121–123, PGT125–128, and PGT135 [91,92]. Current HIV-1 vaccines are not able to induce broadly neutralizing antibodies and only elicit strain-specific neutralizing antibodies. Therefore, chemical and chemoenzymatic synthesis of HIV-1 glycopeptides that mimic the bNAb epitopes but lack the other viral glycoprotein elements has become an important objective in order to characterize the epitopes and to develop new epitope-based HIV vaccines that elicit specific antibodies and thus neutralize in a broad manner.

**Figure 12:**
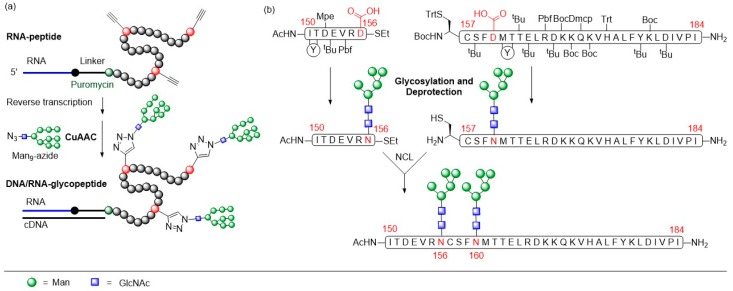
(**a**) Generation of a mRNA-displayed glycopeptide library of ~10^13^ sequences; (**b**) Synthesis of V1V2 glycopeptides using aspartylation and native chemical ligation.

The monoclonal HIV-1 antibody 2G12 recognizes terminal clusters of Man_9_GlcNAc_2_ *N*-glycans on gp120 [93]. In order to generate glycopeptide structures in which the clustering of glycans mimics the 2G12 epitope on gp120, the Krauss group was following an original approach to generate mRNA-displayed peptide libraries of random 33-mer glycopeptides containing 3–5 high-mannose ligands (Figure 12a) [94].

Here, mRNA encoding a library of random peptide sequences was cross-linked to a 3′ puromycin oligonucleotide, which forms a covalent bond to the *C*-terminus of the nascent peptides after ribosomal translation of the mRNAs. Thereby, methionine was substituted by the non-canonical amino acid homopropargylglycine for subsequent ‘click’-glycosylation with Man_9_-azide via CuAAC. The obtained mRNA-displayed glycopeptide library of ~10^13^ sequences was then subjected to ten rounds of selection for binding to 2G12. After each round, peptides that survived selection were amplified by PCR amplification of their cDNA, followed by transcription/translation of the PCR products. In this way, glycopeptides that bind to 2G12 in the low nanomolar to picomolar range were identified.

These candidates were then synthesized in a larger scale using fast flow peptide synthesis with subsequent glycosylation of the alkyne-functionalized peptide backbone via ‘click’ glycosylation [95]. In fast flow peptide synthesis, all reagent solutions also including the activated amino acids are pumped through a heated reactor containing the SPPS resin, thus ensuring a continuous and fresh supply of reagents. Using this method, very short coupling times of 30 s were achieved with peptide yields comparable to microwave-assisted synthesis. The mannosylated glycopeptides were finally coupled to the carrier protein CRM197 and the obtained glycoconjugates were evaluated in binding efficacy studies to 2G12 by using enzyme-linked immunosorbent assays (ELISAs). The glycopeptide conjugates were strongly recognized by antibody 2G12. Rabbit immunogenicity studies of these conjugates to determine their ability to elicit antibody responses with 2G12-like-specificity are currently in progress.

In 2009, the potent monoclonal bNAbs PG9 and PG16 were isolated from an HIV-1-infected patient [96]. Epitope mapping indicated that PG9 and PG16 recognize a different glycan-dependent Env epitope than 2G12 and showed preferred binding towards the trimeric gp120 and gp41 Env complex over the monomeric gp120. Crystal structures of PG9 and PG16 in complex with gp120 V1V2 showed that the antibodies interact with high mannose glycans at N_160_ and N_156_ or N_173_, and an adjacent V1V2 domain consisting of four antiparallel β-strands [97,98]. Whereas, both antibodies required Man_5_GlcNAc_2_ at position 160 to neutralize HIV-1, complex type *N*-glycans at position 156 or 173 play a secondary role for the binding. In terms of vaccine design, a potent gp120-based immunogen could therefore be composed of a Man_5_GlcNAc_2_ moiety at position 160 and a complex *N*-glycan at N156 or N173.

Homogenous gp120 V1V2 35mer glycopeptides carrying *N*-glycans at positions 156 and 160 were synthesized and their binding to mAb PG9 was evaluated by SPR [99]. Due to the steric demands imposed by the close proximity of the glycosylation sites, two separate peptide building blocks were assembled by SPPS and Man_3_GlcNAc_2_ or Man_5_GlcNAc_2_ were coupled to the respective positions by aspartylation (Figure 12b). Subsequently, both glycopeptide building blocks were joined by native chemical ligation (NCL). Binding of the glycopeptide to PG9 was investigated by SPR analysis. Significant binding affinity to the antibody was observed for both ligands with slightly better binding of Man_3_GlcNAc_2_ (K_d_ = 119 nM) over Man_5_GlcNAc_2_ (K_d_ = 311 nM). It was discovered, that the synthetic V1V2 peptides could form disulfide-linked dimers by spontaneous air oxidation. This finding was further investigated by chemical coupling of the synthetic glycopeptides via disulfide bond formation between the cysteines at position 157 under oxidative conditions [100]. Circular dichroism (CD) analysis of the biophysical properties of the V1V2 glycopeptide dimers showed that the random-coiled glycopeptides adopted an ordered β-sheet conformation upon dimerization. The binding of the formed dimers to bNAbs PG9 and CH01 was evaluated by SPR. The binding affinities of both antibodies towards the Man_3_GlcNAc_2_ and Man_5_GlcNAc_2_ glycopeptide dimers were significantly enhanced compared to the binding to the monomeric derivatives. This result indicates that adoption of the β-strand conformation of the V1V2 glycopeptides is required for binding of the antibodies. Crystal structures of PG9 and CH01 in complex with the high-mannose *N*-glycan V1V2 peptides would provide additional information regarding binding preferences.

**Figure 13:**
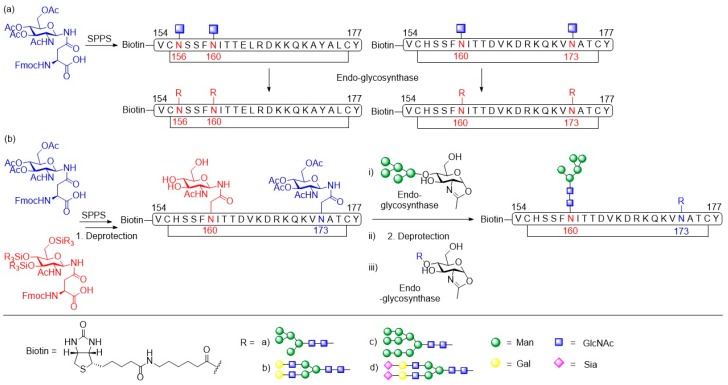
(**a**) Chemoenzymatic synthesis of cyclic gp120 V1V2 peptides carrying different *N*-glycans at positions 156 and 160, and 160 and 173, respectively; (**b**) Convergent chemoenzymatic introduction of two distinct *N*-glycans at positions 160 and 173 in the cyclic gp120 V1V2 peptides.

The Wang group focused on the characterization of the neutralizing epitopes of antibodies PG9 and PG16 by synthesizing V1V2 cyclic glycopeptides (aa154–177) to mimic the gp120 V1V2 epitope and apply them in antibody binding studies [101]. Homogeneous cyclic glycopeptides corresponding to the V1/V2 domain presenting defined *N*-glycans at positions N160 and N156/N173 were prepared by employing a chemoenzymatic method (Figure 13a). Amino acid building blocks glycosylated with a GlcNAc moiety were introduced into the peptide sequence during SPPS. Then, various activated glycan oxazolines were transferred to the GlcNAc moiety by endoglycosidases via a transglycosylation reaction.

The affinities of the glycosylated cyclic peptides towards PG9/PG16 Fab were determined by SPR and ELISA experiments. It was shown that a Man_5_GlcNAc_2_ glycan at the N160 position was essential for PG9 and PG16 recognition. Additionally, a terminal sialylated complex *N*-glycan at glycosylation site N156 or N173 was reported to be important for recognition by PG9 and PG16. A disadvantage of this chemoenzymatic synthesis strategy was the difficulty to generate glycopeptides carrying two or more different glycans since the endoglycosidases usually are unable to distinguish between the GlcNAc acceptors at different glycosylation sites. Therefore, the Wang group optimized the employed method to efficiently and quickly synthesize HIV-1 V1V2 glycopeptides carrying distinct *N*-glycans [102]. This strategy includes the introduction of two orthogonally protected GlcNAc-Asn building blocks into the peptide backbone by SPPS (Figure 13b). After orthogonal deprotection of the GlcNAc residues, site-selective sequential extension of the glycan chains was achieved by glycosynthase-catalyzed transglycosylation reactions.

**Figure 14:**
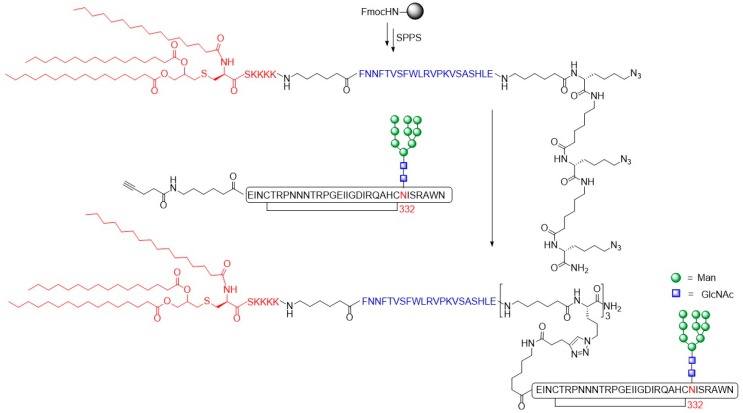
Design of the three-component trivalent HIV-1 vaccine construct.

A number of the bNAbs, including V3-glycan specific antibodies, exhibit preference for the native Env trimer compared to monomeric gp120 [103]. Therefore, multivalent V3 glycopeptides could prove to be efficient to elicit specific bNAbs. The optimized chemoenzymatic method was adopted to prepare a series of HIV-1 V3 glycopeptides [104]. This library was employed to determine the minimal neutralizing epitopes through binding studies with the bNAbs PGT128, PGT121, and 10-1074 that bind to the N332 glycan at the base of the gp120 V3 loop [105,106]. The binding experiments enabled evaluation of the glycan specificity as well as the requirement of the peptide backbone for antigen recognition by these broad neutralizing antibodies. PGT128 was shown to specifically recognize high-mannose glycans within the V3 domain. PGT121 and 10-1074 were specific for a sialylated complex *N*-glycan at the N301 position and a high-mannose *N*-glycan at the N332 glycosylation site, respectively. Based on these findings, a trivalent V3 glycopeptide construct that was expected to exhibited enhanced binding to bNAb 10-1074 was prepared [107]. The afore mentioned chemoenzymatic method [101] was used to synthesize mono- bi- and trivalent 33-mer cyclic gp120 V3 glycopeptide conjugated with Man_9_GlcNAc_2_ *N*-glycans at position N332 to mimic the V3 glycopeptide domains of the Env trimer. Subsequently, the V3 glycopeptides were coupled to a linear bi- or trivalent Lys(N_3_)-functionalized peptide scaffold using copper(I)-catalyzed alkyne–azide 3 + 2 cycloaddition. The binding of the obtained glycopeptide conjugates to the Fab domains of PGT128 and 10-1074 antibodies was evaluated by SPR experiments. Different binding preferences of the PGT128 and 10-1074 antibodies towards the glycopeptides were observed. Whereas PGT128 showed no particular preference for mono-, bi- and trivalent V3 glycopeptides (K_d, mono_ = 3.7 µM, K_d, bi_ = 1.5 µM, K_d, tri_ = 1.1 µM), the 10-1074 antibody showed strongly enhanced binding to the bi- and trivalent V3 glycopeptides in the nanomolar range (K_d, bi_ = 0.31 µM, K_d, tri_ = 0.19 µM) compared to the monomeric derivative (K_d, mono_ = 3.88 µM). The SPR binding results were verified by ELISA analysis. Based on these results, a three-component vaccine construct consisting of trivalent V3 glycopeptides carrying a high-mannose Man_9_GlcNAc_2_ glycan at the N332 site, a T helper epitope peptide derived from tetanus toxin, and the TLR ligand lipopeptide Pam3CSK4 for stimulating immune response was designed (Figure 14) [108]. The cyclic gp120 V3 glycopeptides carrying *N*-glycans at position N332 were synthesized according to the above mentioned protocol. The T cell epitope peptide P30 bearing a *C*-terminal Lys(N_3_) residue for site-specific ligation to the multivalent V3 glycopeptide scaffold via copper (I)-catalyzed alkyne-azide 3 + 2 cycloaddition, and the Pam3CSK4 lipopeptide were assembled by Fmoc-SPPS.

To evaluate the immunogenicity, the trivalent glycopeptide conjugates were incorporated into liposomes and rabbits were immunized without additional adjuvants. In comparison to the previously reported three-component monovalent glycopeptide immunogen that was able to elicit a considerable glycan-dependent antibody response [109], the immunogenicity of the V3 glycopeptide was significantly increased due to multivalent presentation of the ligands. The binding affinities of the antisera to synthetic mono-, bi, and trivalent V3 glycopeptides [107] immobilized on magnetic beads were determined by ELISA. While comparable binding to the mono-, bi-, and trivalent V3 glycopeptides was observed of antisera induced by the monovalent vaccine construct, the antisera induced by the trivalent vaccine construct showed up to 16-fold stronger binding to the multivalent glycopeptides. Unfortunately, neither the antisera induced by the monovalent immunogen nor the antisera induced by the trivalent immunogen showed viral neutralization activity. The authors suggested that these findings could be related to lack of somatic maturation and that further investigations are needed to elicit glycopeptide epitope-specific, broadly neutralizing antibody responses.

The Haynes group also synthesized a homogeneous cyclic V3 glycopeptide bearing Man_9_GlcNAc_2_ glycans at N301 and N332 to target the gp120 V3-glycan epitope [110]. The peptide was assembled using the previously introduced aspartylation/NCL protocol and rhesus macaques were immunized with monomeric Man_9_-V3 glycopeptide formulated in the Toll-like receptor 4 agonist GLA-SE (glucopyranosyl lipid adjuvant-stable emulsion) adjuvant. Isolated serum antibodies were not able to neutralize Env pseudoviruses. The authors concluded that the monomeric V3 glycopeptide was not a potent immunogen and proposed that immunization with a multimerized V3 glycopeptide followed by boosts of sequential Envs may induce potent bNAbs in HIV-1 infected individuals.

**Figure 15:**
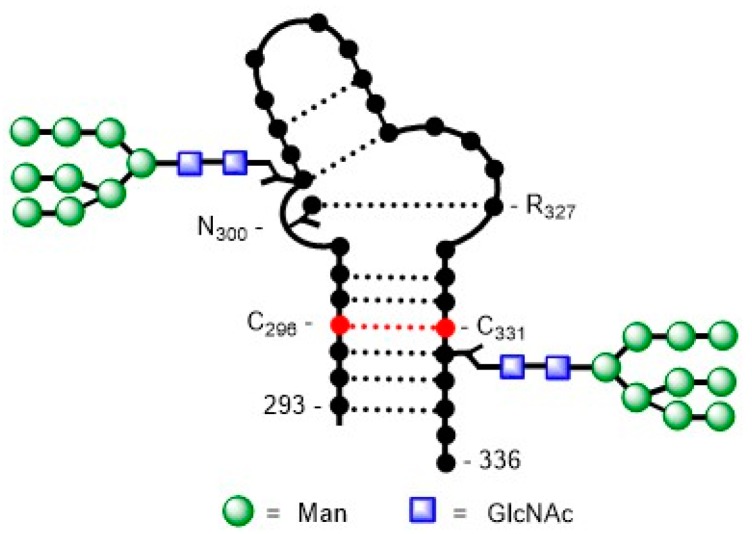
Secondary structure of Man_9_GlcNAc_2_ V3 glycopeptide. Carbonyl or amide functions on the peptide backbone, hydrogen bonds and side chains are by nodes (.), dashed lines and ‘Y’, respectively.

A recent study focuses on the N332 to N334 mutation of the N332 high-mannose glycan on the HIV-1 gp120 V3-loop [111]. The study shows that synthetic V3 glycopeptides bearing a N334 high-mannose Man_9_GlcNAc_2_ glycan were recognized by bNAbs PGT128 and PGT126 but not by 10-1074. Rabbit immunization with a corresponding monovalent three-component glycopeptide immunogen elicited glycan-dependent antibodies with cross-reactivity to different HIV-1 gp120/gp140 glycoproteins carrying N332 or N334 glycosylation sites. These findings indicated that the N334 mutation represents an interesting epitope for further HIV-1 vaccine studies. High-resolution crystal structures can be used to determine differences in selectivity and affinity between closely related broad neutralizing antibodies, leading to a deeper understanding of the roles that somatic mutations might play in the enhancement of binding affinity and neutralization efficacy of bNAbs. A high-resolution crystal structure of the previously described high-mannose V3 glycopeptide carrying Man_9_GlcNAc_2_ glycans at N322 and N301 [110] in complex with the single chain variable fragment (scFv) of the N332-glycan recognizing bNAb DH270.6 was reported [112]. Crystallization experiments showed that the glycopeptide was able to mimic the V3 region of a native-like HIV Env trimer forming a two-stranded β-hairpin with a bulge at the conserved 324GDIR327 motif (Figure 15). Two major regions that participate in antibody–glycopeptide interactions were identified: (i) the Env _324_GDIR_327_ motif, which is an important binding motif for N332-glycan recognizing bNAbs, interacts with the CDRH2 and CDRH3 loops of DH270.6, and (ii) the high-mannose glycan at N332, which is in contact with the CDRH3 and CDRL2 loops of the antibody. Different mutations found in the antibody lineage influenced the binding to the antigen-glycopeptide. Based on these results, the structure of high-mannose V3 glycopeptide can be optimized leading to enhanced affinity and stability of the hairpin.

Consequently, synthetic homogeneous gp120 peptide domains carrying defined glycan structures might be able to function as minimal mimics of epitopes that are recognized by bNAbs and could be used to generate novel HIV-1 vaccines, as they may elicit similar antibody responses to target the HIV Env.

### 3.2. Epstein-Barr Virus (EBV)

Herpesviruses are classified into three primary subfamilies: α, β and γ. The Epstein–Barr virus (EBV), a human gamma-1 herpesvirus, is the cause of lifelong persistent infections and infects the majority of adults (>90%) worldwide [113]. Although most primary infections are asymptomatic, EBV is associated with various cancers [113] and multiple sclerosis (MS) [114] and causes infectious mononucleosis [115], which refers to a group of symptoms such as fever, sore throat, swollen lymph nodes in the neck and armpits, and tiredness. An efficient vaccine is currently not available. The most abundant EBV surface glycoprotein is gp350 which promotes the attachment of the virus to B lymphocytes by binding to the CD21 [116] or CD35 [117] receptors on the surface of B cells facilitating the virus infection. Thus, the EBV gp350 represents a potential target for broad neutralizing antibodies and the determination of the exact epitope is crucial for the development of effective EBV vaccines with the potential to be efficient across multiple strains of EBV.

Recently, the Blixt group performed epitope mapping of *O*-glycosylation sites of the EBV gp350/220 by using synthetic 20mer peptides with a 10mer overlap (ST*HVPT*NLT*A∣ PAS*T*GPT*VS*T*, PAS*T*GPT*VS*T*∣ADVT*S*PT*PAG and so on) covering the entire envelope glycoprotein, and by evaluating auto-induced antibodies from EBV-positive and -negative human sera [118]. The peptides were synthesized by SPPS and immobilized on microarray slides. Subsequent on-chip glycosylation using recombinant GalNAc transferases (GTs) GT 2 and 3, the glycopeptide library was tested in binding assays with EBV-positive and -negative human sera. Binding of several EBV-positive sera to the glycopeptides was observed for peptide sequences 821–841, but not to the corresponding unglycosylated peptides. For further characterization of the epitope properties of peptide 841, glycopeptides with site-specific Tn-glycosylation at position T_845_, T_851_, S_852_ and S_857_, respectively, were prepared and evaluated. Scattered binding preferences of the individual EBV-sera to the particular glycosylation sites was observed. These findings indicate that the identified individual antigenic glycopeptides cannot be used as serological test, but a combination of them generating a unique epitope fingerprint could feature a promising tool to detect EBV-positive sera.

### 3.3. Herpes Simplex Virus (HSV)

The herpes simplex virus (HSV) is a member of the *Herpesviridae* family and is a common, lifelong infection, which often shows no symptoms [119]. HSV is categorized into two types, HSV type 1 (HSV-1) and HSV type 2 (HSV-2), causing oral herpes, ocular infections, encephalitis, and genital herpes, respectively [120]. Whereas HSV-2 is predominantly associated with genital herpes, HSV-1 usually causes orofacial infections but has recently been associated with an increasing number of herpes infections, also including genital herpes.

For correct diagnosis of HSV-1 and HSV-2 infections, the type-specific detection of antibodies in patient sera is very important. Commercial serologic assays that identify HSV-1 and HSV-2-specific antibodies often suffer from cross-reactivity. Therefore, the determination of type-specific peptide epitopes of HSV is of particular interest to develop new strategies for type-specific serology. More than a dozen viral glycoproteins are encoded by the viral genome, including five glycoproteins that are responsible for entry and infection of cells [121].

The Blixt group identified a type-specific glycopeptide epitope of HSV-2 by epitope mapping of the envelope glycoprotein G2 [122]. In a more recent study, this group investigated B-cell epitopes on envelope proteins gB, gC, gD, gE, gG, gH, and gI from HSV-1 and HSV-2 [123]. Synthetic glycopeptides covering the complete sequences of the glycoproteins were prepared by SPPS and immobilized on microarray chips with subsequent on-chip enzymatic glycosylation. The binding of sera from HSV-1- and HSV-2-infected patients to the glycopeptide library was evaluated.

Unique type-specific peptide epitopes were identified and the minimal sero-reactive epitopes were determined. To improve the sensitivity and specificity for HSV serology, two linear HSV-1 and HSV-2 multiepitopes including the respective minimal type-specific epitopes were prepared using microwave-assisted SPPS. The sensitivity and specificity of the synthesized multiepitopes were evaluated using microarray binding assays. Good specificity and sensitivity of the different multiepitopes toward the HSV-type positive sera was observed. These findings demonstrate, that synthetic type-specific glycopeptide epitopes can become an important serodiagnostic tool to diagnose and distinguish between the different HSV types.

### 3.4. Influenza A Virus (IAV)

The Influenza A virus (IAV) is a member of the *Orthomyxoviridae* family and represents one of the most common infectious diseases worldwide leading to high morbidity and mortality. Influenza viruses are the cause of respiratory infection with a variety of symptoms such as sore throat, cough, fever, body aches and gastrointestinal symptoms. In the last decades, the development of anti-influenza agents has become an important objective. These anti-influenza drugs are classified according to their target: the tetrameric transmembrane protein M2 that functions as proton-selective channel and modulates the pH within the virions [124,125], and neuraminidase which is a tetrameric glycoprotein with sialidase activity [126]. In recent years, the homotrivalent hemagglutinin (HA) receptor, which plays a crucial role in the first steps of the viral infection circle, has become a target of interest [127]. HA recognizes and binds terminal sialic acid residues on glycans of epithelial cells. Upon endocytic uptake, the fusion of the endosomal and viral membrane is mediated by a conformational change of the viral hemagglutinin which is triggered by the acidic milieu of late endosomes. Mucins are a class of heavily glycosylated proteins expressed on the surface of epithelial cells or secreted in mucus. Mucin-1 (MUC1) is a transmembrane-bound mucin and is expressed on almost all epithelial tissues. MUC1 is a part of the innate immune system by functioning as barrier against invading pathogens. It has been suggested, that MUC1 is also involved in regulation of inflammation in response to infections [128,129].

**Figure 16:**
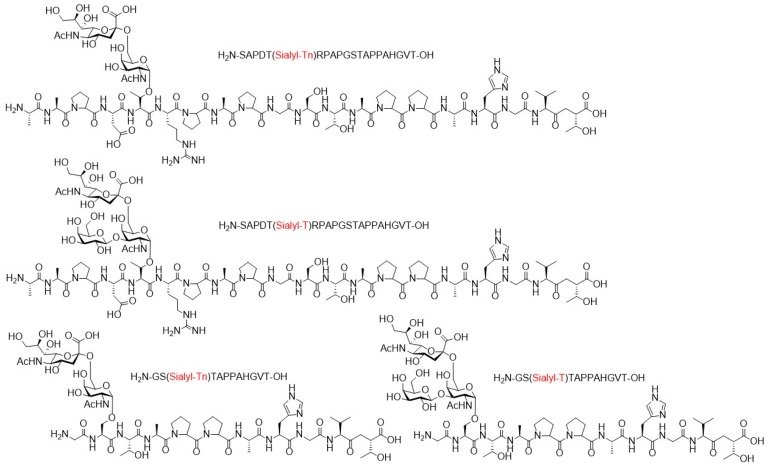
Structures of MUC1 2,6-sialyl-Tn- and 2,6-sialyl-T-antigen peptides.

In the frame of a study from Payne and co-workers, the ability of synthetic MUC1 glycopeptides bearing a sialyl T- or sialyl Tn-antigen to neutralize the influenza virus was investigated by a plaque formation assay in MDCK cells (Figure 16) [130]. While these truncated glycan structures are only weakly expressed by healthy cells, the authors proposed that the α2,6-linked sialic acid residue could mimic the binding epitope of healthy MUC1 towards IAV.

The 20mer MUC1 glycopeptides consisting of the tandem repeat sequence SAPDT*RPAPGSTAPPAHGVT were assembled by SPPS using glycosylserine and threonine Fmoc-building blocks. Additionally, two shorter GS*TAPPAHGVT glycopeptide sequences were generated. The potency of obtained glycopeptides to block infectivity of the virus was determined by a plaque assay in MDCK cells. The assays performed with the MUC1 tandem repeat glycopeptides showed a significant reduction of the number of plaques formed, whereas no neutralization of the virus could be observed with the shorter MUC1 glycopeptide sequences. Furthermore, neutralization of the virus seemed to be independent of the type of glycosylation (sialyl-Tn or sialyl-T antigen) present on the peptide backbone. Based on the results of this study, the authors concluded, that MUC1 does not only act as a virus trap and a ligand for IAV, but also regulates influenza virus-induced inflammation.

### 3.5. Norovirus (NoV)

**Figure 17:**
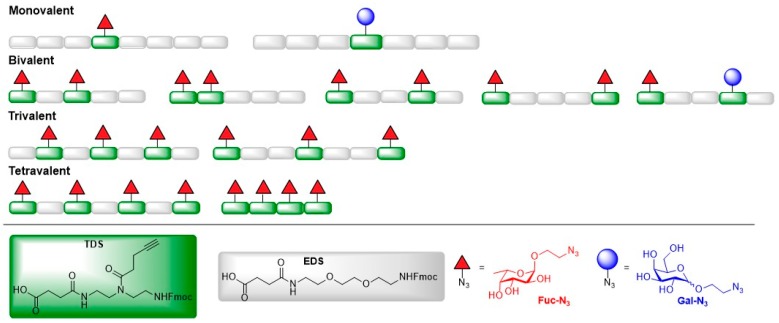
Structure of building blocks TDS and EDS and fucosylated glycooligomers.

Noroviruses (NoVs) are members of the *Caliciviridae* family and are the major cause of nonbacterial gastroenteritis [131]. NoVs are divided into seven genogroups (GI-GVII) which can be further subclassified into various genotypes. The icosahedral virus-like particle (VPL) is formed by self-assembly of 180 copies (90 dimers) of the capsid protein V1 which is again divided into protruding (P) and shell (S) domains [132]. The P domain dimer (P-dimer) contains two binding sites for histo-blood group antigens (HBGAs) which are in addition to α-1,2-linked fucosides common ligands for NoV [133,134]. Recently, two additional α-1,2-linked fucoside binding sites were discovered by X-ray crystallography [135]. So far, knowledge about the mechanism of the ligand-virus interaction is limited and it is not clear, if a multivalent fucose-functionalized ligand simultaneously can bind all four binding pockets. In work from Hartmann et al. multivalent fucosylated precision macromolecules were synthesized according to the previously mentioned protocol [55] and their ability to bind to NoV P-dimers were investigated by native MS, STD NMR, and X-ray crystallography experiments (Figure 17) [136]. Precision glycomacromolecules carrying varying numbers of fucose ligands, and exhibiting different spacing between the ligands on the oligomeric backbone were prepared via SPPoS. The ligand-complex stoichiometry and affinities were obtained in binding assays by using native MS. The parts of the glycooligomers involved in ligand-protein interaction were identified by STD NMR experiments and these findings were further confirmed by X-ray crystallography. No increase in intermolecular complex formation was observed with an increasing number of fucose ligands. Furthermore, no binding of multiple fucose moieties of the same glycooligomers to the fucose binding pockets of NoVs P-dimer could be verified. The authors assumed, that the size of the fucosylated glycomacromolecules inhibits binding to more than one binding site of the P dimer and suggested, that reduction of the hydrodynamic size of the scaffold by using branched instead on linear scaffolds could lead to more promising results.

## 4. Conclusions

Recent developments of new and improved synthetic methodologies have facilitated the preparation of complex and structurally defined glycopeptides and glycopeptide mimetics that can be utilized to probe carbohydrate-pathogen interactions. However, the progression of glycopeptide-based compounds into clinical applications has been limited with respect to proteolytic stability and pharmacokinetics. So far, there are different chemical approaches to increase the proteolytic stability of glycopeptide structures such as the introduction of *C*- or *S*-glycosidic linkages, triazole, oxime or hydrazone ligation, and/or the introduction of unnatural or d-amino acids, or polymeric structures into the peptide backbone without losing the biological function and specificity [137,138]. Additionally, endgroup modification or cyclization of the peptide backbone, or peptide bond substitution can increase the lifetime of the glycopeptide drug [139].

Due to their high polar surface, glycopeptides exhibit a good solubility in aqueous media such as in blood but are also unable to readily cross membrane barriers such as the blood-brain and intestinal barriers. In order to improve these membrane permeabilities, the glycopeptide structure can be either coupled to a lipid carrier or chemically modified by for example acetylation or methylation to increase the lipophilicity [137,139]. Additionally, the glycopeptide drug can be pharmaceutically formulated or delivered using different carrier systems [140]. Chemical and physical degradation of glycopeptide drugs such as deamination, oxidation, denaturation, or aggregation during production and storage are also limiting factors for medicinal applications.

The development of glycopeptide mimetic structures is a promising strategy to come around these challenges. However, the availability of synthetically defined ligands that are close in structure to natural glycopeptide ligands is also critical to investigate and understand the incredible complexity of protein-carbohydrate interactions. Previous extensive studies of E-, P-, and L-selectins and their specific interactions with synthetic glycopeptide ligands have enabled the development of efficient glycomimetic inhibitors such as Rivipansel (GMI-1070) from Glycomimetics Inc. (Rockville, MD, USA), which now is in a phase 3 clinical trial [141,142,143]. Rivipansel is an excellent example that gives hope also for future inhibitor developments involving other lectins and their interactions with glycopeptide or glycopeptide mimetic ligands.

In spite of potential challenges with pharmacokinetic properties, glycopeptide structures linked to immunostimulants have in recent years shown to be highly efficient as potential MUC1 anti-cancer vaccines and a number of candidates are in clinical trials [144,145]. Challenges in this field have been to identify the most optimal antigen structure for immune therapy with respect to the natural glycan microheterogeneity of the protein target and to address the immune system tolerance against the endogenous mucin proteins.

The recent developments of synthetic glycopeptide based anti-adhesive agents, glycan epitopes and (epitope-based) vaccines directed against bacteria and virus infections are described in this review. The applications of such glycopeptide constructs in anti-infectious strategies should be further developed also with regards to the topology of the binding sites of the lectins to increase their specificity and affinity toward the target. Here, multivalent scaffolds presenting the carbohydrate ligands in a well-defined spatial arrangement are powerful tools to overcome the drawback of the relatively weak glycan interactions.

Better understanding of protein glycosylation will help to identify new targets for immunotherapy and accelerate the development of novel serodiagnostic strategies. Based on the studies summarized in this review it can be concluded, that glycopeptides and glycopeptide mimetics have the potency to be utilized in anti-adhesive therapies against viral and bacterial infections, and to mimic the surfaces of viruses and bacteria in order to stimulate the immune systems.
